# *De novo* missense variants in the PP2A regulatory subunit *PPP2R2B* in a neurodevelopmental syndrome: potential links to mitochondrial dynamics and spinocerebellar ataxias

**DOI:** 10.1093/hmg/ddae166

**Published:** 2024-11-20

**Authors:** Priyanka Sandal, Chian Ju Jong, Ronald A Merrill, Grace J Kollman, Austin H Paden, Eric G Bend, Jennifer Sullivan, Rebecca C Spillmann, Vandana Shashi, Anneke T Vulto-van Silfhout, Rolph Pfundt, Bert B A de Vries, Pan P Li, Louise S Bicknell, Stefan Strack

**Affiliations:** Department of Neuroscience and Pharmacology, University of Iowa, Carver College of Medicine, Bowen Science Building, 51 Newton Road, Iowa City, IA 52242, United States; Biologics Discovery, Abbott Laboratories, 100 Abbott Park Road, Abbott Park, IL 60064, United States; Department of Neuroscience and Pharmacology, University of Iowa, Carver College of Medicine, Bowen Science Building, 51 Newton Road, Iowa City, IA 52242, United States; Department of Neuroscience and Pharmacology, University of Iowa, Carver College of Medicine, Bowen Science Building, 51 Newton Road, Iowa City, IA 52242, United States; Department of Molecular Physiology and Biophysics, University of Iowa, Carver College of Medicine, Bowen Science Building, 51 Newton Road, Iowa City, IA 52242, United States; Department of Neuroscience and Pharmacology, University of Iowa, Carver College of Medicine, Bowen Science Building, 51 Newton Road, Iowa City, IA 52242, United States; Department of Neuroscience and Pharmacology, University of Iowa, Carver College of Medicine, Bowen Science Building, 51 Newton Road, Iowa City, IA 52242, United States; PreventionGenetics, Part of Exact Sciences, 3800 S Business Park Ave, Marshfield, WI 54449, United States; Department of Pediatrics, University of North Carolina Chapel Hill, 260 MacNider Hall, CB# 7220, 333 South Columbia St., Chapel Hill, NC 27599-7220, United States; Department of Pediatrics - Medical Genetics, Duke University Medical Center, DUMC Box 103857, Durham, NC 27710, United States; Department of Pediatrics - Medical Genetics, Duke University Medical Center, DUMC Box 103857, Durham, NC 27710, United States; Department of Pediatrics - Medical Genetics, Duke University Medical Center, DUMC Box 103857, Durham, NC 27710, United States; Department of Clinical Genetics, Maastricht University Medical Center, P. Debyelaan 25, Maastricht 6229 HX, the Netherlands; Department^.^ of Human Genetics, Nijmegen Centre for Molecular Life Sciences and Institute for Genetic and Metabolic Disorders, Radboud University Medical Centre, Geert Grooteplein Zuid 10, Nijmegen 6525 GA, the Netherlands; Department^.^ of Human Genetics, Nijmegen Centre for Molecular Life Sciences and Institute for Genetic and Metabolic Disorders, Radboud University Medical Centre, Geert Grooteplein Zuid 10, Nijmegen 6525 GA, the Netherlands; Department^.^ of Human Genetics, Nijmegen Centre for Molecular Life Sciences and Institute for Genetic and Metabolic Disorders, Radboud University Medical Centre, Geert Grooteplein Zuid 10, Nijmegen 6525 GA, the Netherlands; Division of Neurobiology, Department of Psychiatry and Behavioral Sciences, Johns Hopkins University School of Medicine, 600 N Wolfe St, CMSC 8-117, Baltimore, MD 21287, United States; Department of Biochemistry, University of Otago, 710 Cumberland St., Dunedin North, Dunedin 9016, New Zealand; Department of Neuroscience and Pharmacology, University of Iowa, Carver College of Medicine, Bowen Science Building, 51 Newton Road, Iowa City, IA 52242, United States; Iowa Neuroscience Institute, Intellectual and Developmental Disabilities Research Center, University of Iowa, Carver College of Medicine, Iowa City, IA 52242, United States

**Keywords:** neurodevelopmental disorders, cerebellar ataxias, protein phosphatase 2A, mitochondrial dynamics, dynamin-related protein 1

## Abstract

The heterotrimeric protein phosphatase 2A (PP2A) complex catalyzes about half of Ser/Thr dephosphorylations in eukaryotic cells. A CAG repeat expansion in the neuron-specific protein PP2A regulatory subunit *PPP2R2B* gene causes spinocerebellar ataxia type 12 (SCA12). We established five monoallelic missense variants in *PPP2R2B* (four confirmed as *de novo*) as a cause of intellectual disability with developmental delay (R149P, T246K, N310K, E37K, I427T). In addition to moderate to severe intellectual disability and developmental delay, affected individuals presented with seizures, microcephaly, aggression, hypotonia, as well as broad-based or stiff gait. We used biochemical and cellular assays, including a novel luciferase complementation assay to interrogate PP2A holoenzyme assembly and activity, as well as deregulated mitochondrial dynamics as possible pathogenic mechanisms. Cell-based assays documented impaired ability of *PPP2R2B* missense variants to incorporate into the PP2A holoenzyme, localize to mitochondria, induce fission of neuronal mitochondria, and dephosphorylate the mitochondrial fission enzyme dynamin-related protein 1. AlphaMissense-based pathogenicity prediction suggested that an additional seven unreported missense variants may be pathogenic. In conclusion, our studies identify loss-of-function at the *PPP2R2B* locus as the basis for syndromic intellectual disability with developmental delay. They also extend *PPP2R2B*-related pathologies from neurodegenerative (SCA12) to neurodevelopmental disorders and suggests that altered mitochondrial dynamics may contribute to mechanisms.

## Introduction

Intellectual and developmental disabilities are estimated to affect about 2% of individuals by mostly unknown etiologies [1]. The recent advances in genome/exome sequencing have provided a powerful tool to discover insights into numerous disease-causing mutations. A large-scale study demonstrated that about half of severe developmental disabilities are the result of *de novo* mutations [2]. An exome sequencing study performed on 41 probands and their parents by Hamdan *et al*. identified the possibly pathogenic variant R149P in the *PPP2R2B* gene as associated with intellectual disability and neurodevelopmental delay [3]. *PPP2R2B* encodes a regulatory subunit of the protein phosphatase 2A (PP2A, PPP2) trimeric complex. PP2A is a ubiquitously and highly expressed serine/threonine protein phosphatase complex made up of a variable regulatory B subunit that interacts with a scaffolding A subunit and catalytic C subunit. Together with protein phosphatase 1, PP2A catalyzes the majority of Ser/Thr dephosphorylation in eukaryotic cells. PP2A regulatory subunits are encoded by three gene families (*PPP2R2*, *PPP2R3*, and *PPP2R5*), with each family containing three to five genes. While PP2A scaffolding and catalytic subunits are essential and ubiquitously expressed, tissues and cell types express their own complement of regulatory subunits that dictate PP2A subcellular localization, substrate specificity, and regulation by posttranslational mechanisms [4, 5]. PPP2R2B (a.k.a Bβ, B55β) is one of three regulatory subunits selectively expressed in the nervous system. The best characterized isoform of *PPP2R2B* is the mitochondria-targeted Bβ2 that localizes the PP2A complex to the outer mitochondrial membrane to mediate mitochondrial fission by dephosphorylating and activating the large GTPase dynamin-related protein 1 (Drp1) [6–8].

Drp1-dependent mitochondrial fission is opposed by mitochondrial fusion and together they are critical for mitochondrial quality control, bioenergetics, and cell differentiation, proliferation, and survival [9–11]. Indeed, mice with selective KO of the Bβ2 isoform of *PPP2R2B* display an allele dose-dependent increase in the length of neuronal mitochondria and protection from ischemic stroke [12].

In the present study, we newly identify four individuals with a neurodevelopmental disorder (NDD) and with monoallelic *PPP2R2B* variants (three confirmed *de novo*). Together with the previously reported R149P *de novo* variant [3], we examined the five variants’ pathogenic mechanisms, focusing on PP2A holoenzyme formation, mitochondrial dynamics, Drp1 dephosphorylation, and neuronal survival. We discovered partial to complete loss-of-function of these missense variants, suggesting that *PPP2R2B*-related NDDs may be caused by dysregulation of the mitochondrial fission/fusion equilibrium.

## Results

### Phenotypes of individuals with monoallelic *PPP2R2B* variants

We studied five *PPP2R2B* variants (reference: NM_181675.4) identified in patients with NDD. The substitution variant p.Arg149Pro (R149P) was previously reported as *de novo* in an individual with moderate intellectual disability, pervasive developmental delay, along with intractable seizures, autistic features, and aggressive behaviour [3]. We identified four additional individuals with NDD carrying the monoallelic variants p.Thr246Lys (T246K), p.Asn310Lys (N310K), p.Glu37Lys (E37K), and p.Ile427Thr (I427T) by whole exome sequencing (Table 1). Trio sequencing confirmed N310K, E37K, and I427T as *de novo* variants; the inheritance of T246K is unknown. I427T was associated with an additional monoallelic variant in *TAOK1* (p.Arg562^*^, reference: NM_020791.4) that was inherited from the phenotypically unremarkable father. According to gnomAD (v4.1), *TAOK1* (like *PPP2R2B*) is intolerant to loss-of-function (pLI = 1.00). However, inheritance of loss-of-function *TAOK1* alleles has previously been documented, suggesting incomplete penetrance [13]. The patient with the *PPP2R2B* E37K variant carried a second, *de novo* variant in *RNU4-2* (n.64_65insT), which is associated with a recently described neurodevelopmental syndrome [14].

**Table 1:** Clinical presentation of individuals affected by *PPP2R2B* variants. Information is incomplete or redacted for patient 3 due to lack of consented follow-up.

	**Patient 1**	**Patient 2**	**Patient 3**	**Patient 4**	**Patient 5**
**Reference**	Hamdan *et al*. [3]	this report	this report	this report	this report
**Variant** **(NM_181675.4)**	c.413G>C; p.(R149P)	c.737C>A; p.(T246K)	c.930 C>A; p.(N310K)	c.109G>A; p.(E37K)	c.1280T>C; p.(I427T)
**ClinVar accession**	2 503 290	1 712 310	1 319 957	SUB14519904	1 711 732
**Inheritance**	de novo	unknown	de novo	de novo	de novo
**Sex**	male	female		male	female
**Age at last assessment**	7 yr	18 yr		6 yr	3 yr 10 mo
**Motor activity**	normal but delayed fine motor	broad-based gait; gait imbalance; stereotypical body rocking; dyskinesia; tremors; jerky eye and jaw movements; hypotonia		Hypotonia; delayed (walking at 4 yr); stiff gait	broad-based gait (not progressive); tremors at rest and with intention; hypotonia
**Epilepsy**	intractable; focal tonic seizures developed into myoclonic, astatic seizures (Doose Syndrome); cluster of seizures at 2–3 months until 6 y. EEG: generalized polyspike activity, now normal	hyperkinetic seizures; myoclonus; normal EEG		tonic–clonic seizures treated with Keppra	history of afebrile seizures; EEG (6y): abnormal left posterior head region spike and slow waves
**Intellectual disability**	moderate	yes		yes	moderate
**Autism spectrum**	not noted	yes		not noted	not noted
**Developmental delay**	yes	yes		yes	yes
**Language**	delayed	speech articulation difficulties		delayed; recognizable words at 6 years	delayed; ongoing issues with articulation
**Affect and mood**	aggressive, on aripiprazole and guanfacine	abnormal behavior; aggressive; anxiety; ADHD		normal	some aggressive behavior (when menstruating); oppositional behavior; ‘high energy’ but no ADHD diagnosis
**Sensory abnormalities**	not noted	not noted		hearing loss L > R; cerebral visual impairment; nystagmus	left auditory neuropathy (uses hearing aid)
**Head/Cranium/MRI**	normocephalic; normal MRI	normocephalic; normal MRI		microcephaly, MRI: thin corpus callosum, prominent liquor spaces, low-lying cerebellar tonsils, delayed myelination	microcephaly, hydrocephalus (resolved); MRI (11 mo): increased subarachnoid spaces and borderline ventricles; thin corpus callosum. 10 y: unremarkable

**Figure 1:**
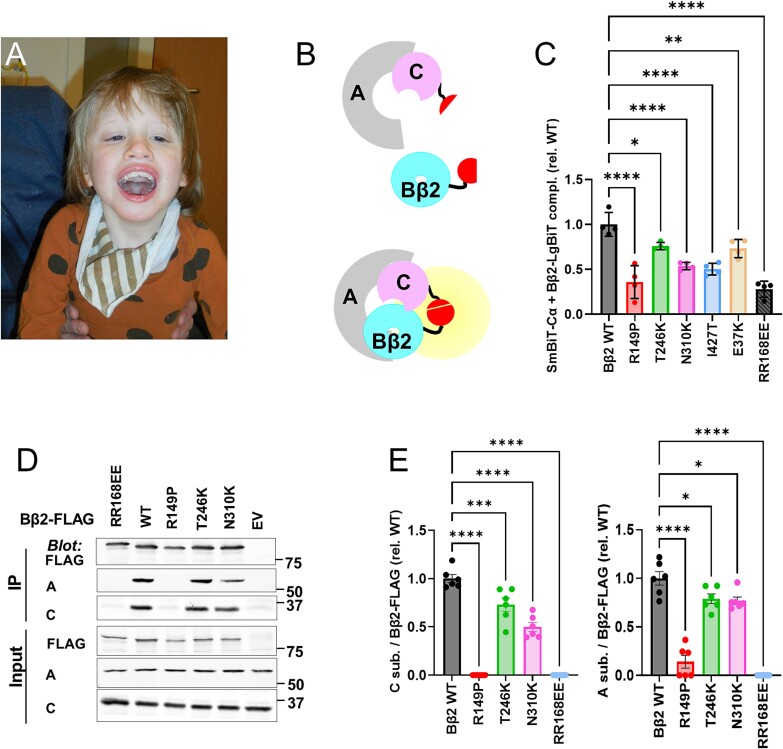
Effect of *PPP2R2B* variants on PP2A holoenzyme assembly. (A) Facial photograph of patient 4 showing dysmorphic features. (B and C) A novel NanoBiT luciferase complementation assay (principle in (B)) shows significantly weakened interactions of all phenotypically characterized PPP2R2B/Bβ2 variants with the catalytic Cα subunit in transfected COS-1 cells; RR168EE is a mutation at the interface to the A subunit known to disrupt the PP2A complex. Data points are means of four independent experiments with 6 technical replicates each. (D and E) Co-immunoprecipitation of FLAG-GFP tagged Bβ2 variants from COS-1 cells shows that all variants associate with less endogenous A and C subunits compared to wild-type (WT) Bβ2. (E) Data points are normalized to C and A subunit levels in the co-IP from 6 independent experiments. Molecular mass markers are shown in kDa on the right of the representative immunoblot in (D). Error bars show means ±95% CI. Asterisks denote significant differences compared to WT based on one-way ANOVA analysis with Dunnett’s multiple comparisons *post-hoc* test. ^*^P < 0.05; ^*^^*^P < 0.01; ^*^^*^^*^P < 0.005; ^*^^*^^*^^*^P < 0.0001.

All five probands were diagnosed with intellectual disability and developmental delay. Shared with other intellectual disability syndromes, other common diagnoses included various motor symptoms (4 probands), language and articulation delay (4), seizures (4), aggressive behavior (3), and hypotonia (3). Hearing loss/auditory neuropathy were noted in probands 4 and 5. Both probands were also diagnosed with resolved microcephaly. Proband 4 was diagnosed with cerebral visual impairment and nystagmus, whereas proband 5 had a rudimentary uterus and absent right kidney. Of note, only incomplete clinical information is available for patient 3 (N310K), because their caregivers did not participate in follow-ups (Table 1). A facial photograph of patient 4 shows the following dysmorphologies: down-slanting palpebral fissures, short filtrum, broad mouth with full lips, irregular position of some teeth, and flat but normally closed palate (Fig. 1A).

### Characteristics of PPP2R2B missense mutations

PP2A subunits, including the PPP2R2 family of regulatory subunits are highly conserved in metazoans (see sequence alignment of human PPP2R2A/B/C/D and their orthologues from *Drosophila melanogaster* and *Caenorhabditis elegans*, Supplemental Fig. S1). Residues affected by clinically characterized missense mutations are either phylogenetically invariant (I427), highly conserved (E37, T246, N310), or moderately conserved (R149). Of note, proline substitutions (as in R149P) tend to be pathogenic because they disrupt secondary structures [15]. Mapping the mutated residues onto a structure model of the Bβ2 isoform of PPP2R2B generated by AlphaFold2 reveals that E37, N310 and R149 are surface-exposed, whereas T246 and I427 are buried within the 7-bladed β-propeller of the structure. Neither residue is part of an interface between the scaffolding A or the catalytic C subunit of PP2A; however, the R149P missense mutation may influence the structure of a β-hairpin that makes multiple contacts with the A subunit (Supplemental Fig. S2A and B).

While all known missense variants map to the common β-propeller domain, *PPP2R2B* is diversified by alternative splicing and promoter use at its N-terminus. In humans, 5 N-terminal coding variants have been annotated that range between 442 and 509 residues in length (Supplemental Fig. S2C). We have shown that the variable N-termini of the two major isoforms, Bβ1 and Bβ2, target the PP2A holoenzyme to the cytosol and outer mitochondrial membrane, respectively [7]. Because little is known about the biological function of the most common reference variant Bβ1, we characterized NDD mutations in the context of the outer-mitochondrial Bβ2 while numbering residues according to ClinVar and the reference variant (Bβ1).

### Effects of NDD mutations on PP2A holoenzyme incorporation and activity

We developed a novel NanoBiT split luciferase complementation assay to investigate mutant PPP2R2B association with the A and C subunit in intact cells (Fig. 1B). We first optimized the assay by comparing complementation between Bβ2 and Cα subunits tagged at either the N- or C-terminus with either the small (SmBiT) or large (LgBiT) fragment of NanoLuc. Aα without Lg/SmBiT fusions was co-transfected into COS-1 cells for stoichiometric expression of all three PP2A subunits. C-terminally LgBiT-tagged Bβ2 yielded the highest luminescence when combined with N-terminally SmBiT-tagged Cα. This combination also minimized complementation with a previously described Bβ2 mutant that does not interact with the PP2A complex (A/B interface mutation RR168EE [16, 17]). All five clinically phenotyped *PPP2R2B* variants showed highly significant impairments in this *in situ* PP2A holoenzyme incorporation assay, with the R149P substitution reducing binding to the levels of the RR168EE negative control (Fig. 1C).

We next tested a subset of the clinically described *PPP2R2B* variants (R149P, T246K, N310K) by binding of FLAG-tagged Bβ2 to endogenous A and C subunits. According to FLAG co-immunoprecipitation from transfected COS-1 cells, all variants displayed significantly reduced binding to endogenous A and C subunits, with R149P binding at background levels (Fig. 1D and E).

To directly test phosphatase activity of mutant PPP2R2B-associated PP2A, Bβ2-GFP complexes were isolated from transfected COS-1 cells with anti-GFP nanobodies and subjected to in vitro phosphatase assays. Phosphatase activity corresponded to the level of the PP2A catalytic subunit in the coIP and was significantly reduced by the R149P variant (Supplemental Fig. S3).

### Variants accelerate PPP2R2B protein turn-over

**Figure 2:**
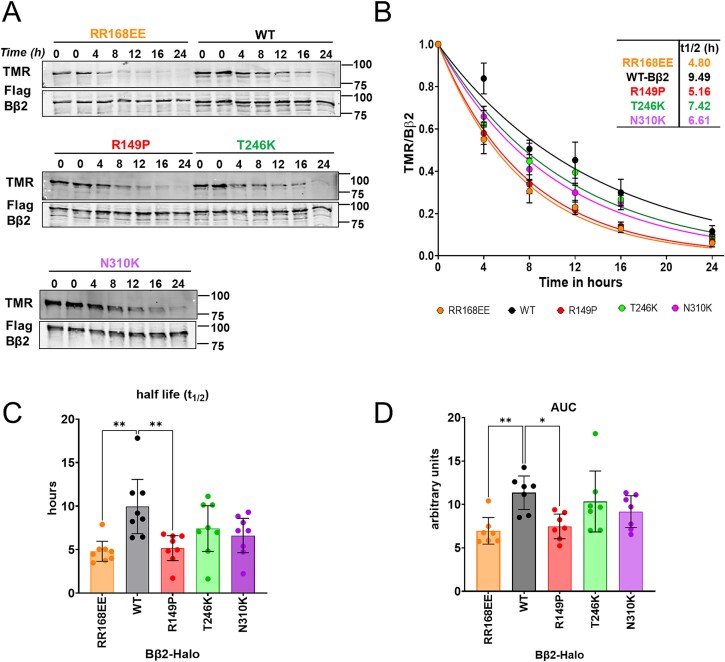
The R149P variant promotes PPP2R2B/Bβ2 degradation. (A) Representative blots of a Bβ2-HaloTag protein turn-over time course. COS-1 cells were transiently transfected with Bβ2 WT or variants that are C-terminally-fused with FLAG and HaloTag subunits. Cells were then pulse-labeled with TMR fluorescent ligand (30 min, 50 nM) followed by chase with media containing 7-bromoheptanol (10 μM) for the indicated times. Following SDS-PAGE of total lysates, a decay in TMR fluorescence was detected by infrared imaging and FLAG was detected for total protein by immunoblotting of the same blot. Molecular weight markers (in kDa) are indicated on the right of the blots. (B–D) Bβ2 degradation over time was quantified as the ratio of TMR to total Bβ2-FLAG-HaloTag signal and further normalized to time zero (no chase). Data points in (B) are means ± SEM from 6–7 independent experiments. (C) Half-lifes (t_1/2_) were calculated from fitting the normalized data points to a one-phase decay curve. (D) Area-under-the curve was calculated from the same curves. Data points in (C and D) represent individual experiments and error bars represent means ±95% CI. ^*^P < 0.05; ^*^^*^P < 0.01 according to one-way ANOVA with Dunnett’s *post hoc* test.

Monomeric PPP2R2-family regulatory subunits are rapidly degraded by the ubiquitin-proteasome pathway [16]. To gain independent evidence that NDD-associated *PPP2R2B* variants destabilize interactions with the A and C subunits in the PP2A heterotrimer, we conducted pulse-chase experiments with Bβ2-HaloTag fusion proteins uniquely capable of measuring the turn-over of long-lived proteins [18]. Briefly, COS-1 cells were transfected with either Halo-tagged Bβ2 WT or mutant plasmids. After pulse labeling with the fluorescent tetramethylrhodamine (TMR)-HaloTag ligand, labeling of newly synthesized Bβ2 was chased by 7-bromoheptanol for up to 24 h. The fluorescence signals were normalized to total Bβ2 protein levels detected by western blotting (Fig. 2A). Turn-over rates (t_1/2_) were determined by monophasic decay curve fits (Fig. 2B and C). Compared to wild-type (WT, 9.5 h) Bβ2, the R149P variant and the known monomeric mutant (RR168EE) conferred significantly faster turn-over rates (4.8 and 5.2 h, respectively). The T246K and N310K mutations accelerated Bβ2 turn-over partially, but not significantly (7.4 and 6.6 h, respectively), in keeping with their reduced, but not absent binding to the A and C subunit of the PP2A holoenzyme (Fig. 1C and D). Decay time courses were also quantified by area-under-the-curve (AUC), yielding similar levels of significance (Fig. 2D). These experiments indicate that NDD-associated PPP2R2B variants are preferentially targeted for degradation.

### Impact of PPP2R2B variants on mitochondrial localization

**Figure 3:**
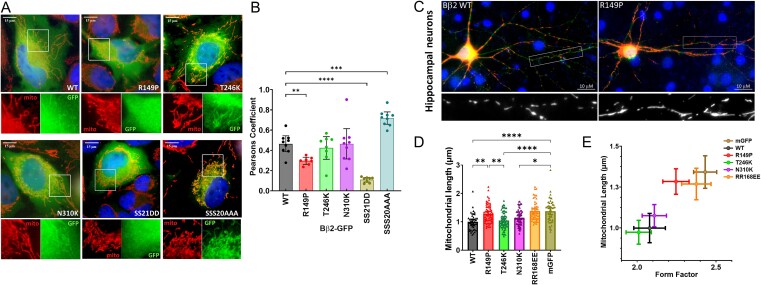
PPP2R2B/Bβ2 variant effect on mitochondrial localization and fission. (A and B) Bβ2-GFP expressed HeLa cells (green, representative images in (A)) were colocalized with mitochondria (COX2 immunofluorescence, red) and colocalization was quantified as Pearsons coefficient (B). Bβ2 SS21DD and SSS20AAA are previously described mutants with decreased and increased mitochondrial localization, respectively [8]. Data points are the means of 7-9 independent experiments (~30–40 cells per transfection). Error bars are 95% CI. (C–E) Primary rat hippocampal neurons were cotransfected with Bβ2-GFP and or outer-mitochondrial GFP (mGFP, green) together with Mito-V5-mRFP (red) at 4:1 mass ratios and fixed at 12 days-in-vitro followed by immunofluorescence imaging (representative images in (C)). (D) Data points are mean mitochondrial length (μm) of 43–67 neurons from four separate transfections and two primary cultures (error bars are means ±95% CI). (C) An XY-plot correlates mitochondrial length and form factor, two measures without common parameters, from the same set of neurons (means ±95% CI). ^*^P < 0.05; ^*^^*^P < 0.01; ^*^^*^^*^P < 0.001; ^*^^*^^*^^*^P < 0.0001 by one-way ANOVA followed by Dunnett’s *post hoc* test compared to WT (B) or Tukey’s *post hoc* test (C).

The alternative N-terminus of Bβ2 mediates PP2A localization to the OMM via interaction with the translocase of the outer mitochondrial membrane (TOM) complex [6, 7]. Phosphorylation of Ser20,21,22 in the targeting sequence antagonizes this interaction, rendering Bβ2 cytosolic, whereas auto-dephosphorylation of these sites restores Bβ2’s OMM localization [8]. A corollary of these findings is that OMM localization of Bβ2 requires incorporation into an active PP2A complex. To test this hypothesis, we quantified colocalization of Bβ2-GFP with immunofluorescently labelled mitochondria in transfected HeLa cells, a flat cell line amenable to epifluorescence imaging. Pseudo-phosphorylated (SS21DD) and non-phosphorylatable (SSS20AAA) Bβ2 served as controls, displaying respectively, largely cytosolic (Pearson’s Coefficient, PC ≈ 0) and mitochondrial localization (PC ≈ 1) compared to WT Bβ2 (PC ≈ 0.5, Fig. 3A and B). The R149P variant significantly reduced Bβ2 targeting to mitochondria, implying that this variant does not recruit sufficient PP2A activity to overcome phosphorylation at Ser20-22. No significant effects of the T246K and N310K variant on Bβ2 localization to mitochondria were observed.

### Bβ2 variants influence mitochondrial shape in primary neurons

Overexpression of Bβ2 promotes mitochondrial fission, requiring mitochondrial localization and ability of Bβ2 to incorporate into the PP2A holoenzyme [7]. On the other hand, global Bβ2 knockdown or knock-out induces mitochondrial elongation by unopposed fusion in neurons but not in glia [12, 19]. We therefore tested whether Bβ2 variants retain the ability to drive mitochondrial fission in primary hippocampal neurons. Compared to transfection of an OMM-targeted GFP control, Bβ2-GFP significantly reduced the length and form factor of mitochondria labelled with mito-mRFP (Fig. 3C–E). The known monomeric RR168EE mutation or the R149P variant in contrast nullified the effect of Bβ2 on mitochondrial shape. However, we did not detect an effect of the T246K and N310K variant on Bβ2 activity in the context of this assay.

### Differential effects of Bβ2 variants on neuronal survival

**Figure 4:**
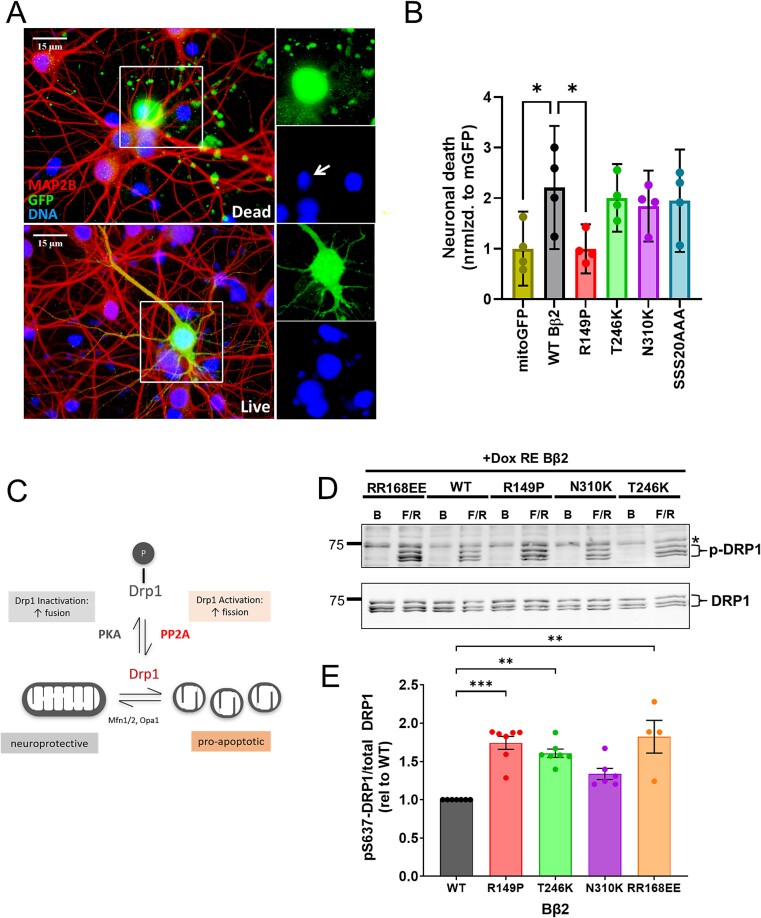
Effects of PPP2R2B/Bβ2 variants on neuronal survival and Drp1 dephosphorylation. (A and B) Primary hippocampal neurons were transfected with Bβ2-GFP WT/variant or outer-mitochondrial (Mito)-GFP negative control at 9 days-in-vitro. 72 h post-infection, cultures were fixed and transfected (GFP-positive) neurons were scored as dead or alive based on nuclear size/morphology (Hoechst stain), and integrity and MAP2B staining of GFP-positive neurites. Representative images are shown in (A); the arrow points to a condensed, apoptotic nucleus. (B) Quantification of neuronal death (dead/(dead + live) GFP-positive neurons; 200–300 neurons per experiment) in 4 independently prepared cultures normalized to the Mito-GFP negative control. (C) Model of regulation of Drp1 activity and mitochondrial fission by phosphorylation/dephosphorylation of the inhibitory Ser637 site. PP2A/Bβ2 and PKA/AKAP1 activity antagonize each other in the control of mitochondrial fission. (D and E) Cell-based Drp1 dephosphorylation assay utilizing HEK293 cells in which the majority of PP2A holoenzymes is replaced by a defined PP2A/Bβ2 holoenzyme [17]. Drp1 is largely dephosphorylated under basal (b) conditions; the asterisk indicates a non-specific band. However, upon forskolin/rolipram treatment (F/R, 20/2 μM for 20 min), pSer637-Drp1 is detectable reflecting the balance of PKA and PP2A/Bβ2 WT/variant activities (D). (E) Quantification of pSer637 to Drp1 ratios normalized to WT from 4–6 independent experiments. ^*^P < 0.05; ^*^^*^P < 0.01; ^*^^*^^*^P < 0.001; either by one-way ANOVA followed by Dunnett’s *post hoc* test (B) or by Kruskal-Wallis test with Dunn’s post hoc test (E).

Prolonged over-expression of Bβ2 in hippocampal cultures induces neuronal apoptosis, again requiring the regulatory subunit to associate both with the TOM complex and with the rest of the PP2A holoenzyme [19]. We therefore asked whether NDD-associated variants modulate the pro-apoptotic activity of PP2A/Bβ2 in primary hippocampal neurons. Three days after transfection at 6 days-in-vitro, cultures were fixed and GFP-positive neurons were scored for fragmented neurites, loss of MAP2B, and apoptotic/pycnotic nuclei. Expression of WT, T246K, and N310K Bβ2 promoted apoptosis compared to mitoGFP, while the R149P variant and the negative control (RR168EE) did not (Fig. 4A and B). This again indicates that R149P is a loss-of-function variant.

### Drp1 dephosphorylation by Bβ2 variants

PP2A/Bβ2 promotes mitochondrial fission by dephosphorylating an inhibitory protein kinase A (PKA) phosphorylation site on Drp1, Ser637 (Fig. 4C) [8]. We asked whether decreased ability to dephosphorylate Drp1 underlies abnormal modulation of mitochondrial fission and apoptosis by Bβ2 variants. To this end, we generated “PP2A reduction” (RE) HEK293-TRex cell lines in which ~50%–60% of endogenous PP2A holoenzymes are replaced by a single PP2A holoenzyme using a combination of RNAi and doxycycline-inducible expression of charge-reversal pairs of A and B subunits that bind to each other, but not to endogenous A and B subunits [17]. Replacement of endogenous B subunits with Bβ2 WT or NDD-variants resulted in no net change in A, B, and C subunit abundance, and WT and variants were expressed at similar levels (Supplemental Fig. S4). To assess Drp1 Ser637 dephosphorylation, cells were stimulated with the adenylate cyclase agonist forskolin and the phosphodiesterase inhibitor rolipram to activate PKA for 20 min before lysis and immunoblotting for phospho-Ser637-Drp1 and total Drp1. PP2A/Bβ2 WT dephosphorylated Drp1 best (lowest pSer637-Drp1 to total Drp1 ratio), followed by T246K Bβ2 (but not significantly different from WT), N310K Bβ2 (*P* < 0.01 from WT), and R149P Bβ2 (*P* < 0.001 from WT) (Fig. 4D and E). This rank order corresponds to the rank order for association of variants with the endogenous PP2A holoenzyme (Fig. 1C and D).

### Predicted prevalence of *PPP2R2B*-related NDDs

To this end, we cross-referenced ClinVar with gnomAD (v.4.1.0), filtering for *PPP2R2B* missense variants with an allele frequency of less than 10^−4^. The resulting 27 variants were analyzed by AlphaMissense, a recently developed deep learning algorithm built on AlphaFold2 structure prediction [20]. The five variants described in this report received a pathogenicity score greater than 0.5 (from N310K = 0.527 to R149P = 0.989). Again, AlphaMissense pathogenicity scores paralleled the variants’ impairments in our biochemical assays. Most importantly, however, this *in silico* analysis identified an additional seven unreported, but probably pathogenic missense variants in *PPP2R2B*, with diagnoses of SCA12 (2) and inborn genetic diseases (3) (Supplemental Table S1**,**  Supplemental Fig. S1).

## Discussion

This report highlights five individuals with *PPP2R2B*-related NDD, adding to and including the one individual previously reported with similar features [3]. In all but one of these individuals, inheritance could be confirmed as *de novo*, reinforcing the likely pathogenicity of these missense variants. Common features include cardinal features of NDD, such as intellectual disability, developmental including speech delay, and seizures. Broad-based or stiff gate observed in three probands are suggestive of early onset ataxia. *In silico* analysis confirmed likely pathogenicity and pointed to at least an additional seven previously unreported variants as causative for *PPP2R2B*-related NDD with ataxic features.

Two individuals with *de novo PPP2R2B* variants received dual diagnoses: I427T with a paternally inherited *TAOK1* variant and E37K with a *de novo RNU4-2* variant. Given the heterogeneity and overlap in the presentation of associated symptoms, it is currently not possible to determine the relative contribution of the dual variants to pathogenicity.

Cellular characterization of these five missense *PPP2R2B* variants revealed significantly weakened interactions with the common A and C subunits of the PP2A heterotrimer. To arrive at this conclusion, we developed a novel luciferase complementation assay that quantifies PP2A holoenzyme assembly in intact cells. This robust and scalable assay may be useful for preliminary classification of new PP2A variants (or subunits of other protein complexes) as they emerge from large-scale gene discovery.

Three of these five *PPP2R2B* variants were further analyzed for mitochondrial localization, induction of neuronal cell death, and dephosphorylation of the mitochondrial fission enzyme Drp1, revealing partial to complete loss-of-function compared to a known monomeric mutant. The *PPP2R2B* gene is highly intolerant to loss-of-function variants in normal individuals (pLI = 1.00, gnomAD v4.1). This indicates that *PPP2R2B*-related NDD is caused by loss-of-function, perhaps haploinsufficiency, potentially involving dysregulation of the mitochondrial fission/fusion equilibrium.

PPP2R2B targeting to subcellular regions and substrates is governed by alternative splicing and promoter use of its N-terminal alternative sequences, giving rise to 6 documented coding isoforms in humans. However, the five missense variants studied here map to the common β-propeller domain at the C-terminus of the protein. The cytosolic Bβ1 and the mitochondrial Bβ2 isoforms are the only isoforms conserved in the vertebrate subphylum, and they are most abundant in the human brain (Supplemental Fig. S2C). We focused our analysis on NDD missense variants in the context of the Bβ2 isoform because we know of its subcellular (mitochondrial) localization, one of its substrates (Drp1), and many of its physiological roles based on KO studies in mice [12]. On the other hand, Bβ1 mRNA is expressed earlier than Bβ2 mRNA in the developing rat brain [7], thus providing Bβ1 variants with advanced opportunities to derail normal brain development. Whether *PPP2R2B*-related NDD is attributable to aberrant dephosphorylation of substrates of PP2A/Bβ1 or PP2A/Bβ2, or both, is an important question that requires further studies.

Another salient question concerns the relationship between *PPP2R2B*-related NDD and SCA12, which is caused by a CAG trinucleotide repeat expansion in *PPP2R2B*. SCA12 is a rare autosomal dominant spinocerebellar ataxia with an onset of symptoms between 8 and 55 years (median in the fourth decade). SCA12 presents with action tremor but only mild cerebellar ataxia. Extrapyramidal symptoms and dementia have also been reported [21, 22].

Suggestive of a link with SCA12, individuals with *PPP2R2B* missense variants present with motor delay and/or broad-based gait (Table 1). While the CAG repeat is not part of any of the coding regions of the four *PPP2R2B* isoforms expressed in the human brain, it is present in the 5′ UTR of the major 203 isoform [ENST00000394411.9] encoding Bβ1. The repeat is 229 bases upstream of the transcription initiation site of the 202 isoform [ENST00000394409.7], which also encodes Bβ1, suggesting that the expansion could modulate transcription or mRNA stability of about half of *PPP2R2B*-derived transcripts [22, 23]. Transcription initiation sites of other *PPP2R2B* isoforms, including that of the Bβ2-encoding 201 [ENST00000336640.10], are more than 177 kb upstream of the CAG repeat, making it unlikely that a repeat expansion could influence their expression.

There is an increasing recognition of the link between neurodevelopmental and neurodegenerative disorders. For instance, missense pathogenic variants in the PP2A regulatory subunit *PPP2R5D* causing the NDD Houge-Janssens Syndrome 1 (MIM: 616355) [24] have recently been associated with early-onset Parkinsonism [25–27]. While the present report suggests that *PPP2R2B*-related NDD are similarly associated with SCA12, this link remains to be further explored.

## Materials and methods

### Antibodies and reagents

The following commercially available antibodies were used in this study: mouse anti-PP2A/C (BD Biosciences cat # 610556), rat anti-PP2A-A (clone 6G3, Cell Signaling cat # 2260), rabbit anti-PP2A/B’δ (Abcam cat # ab188323), rabbit anti-PP2A-B’ε (Abcam cat # ab1985000), mouse anti-DLP1 (Drp1, BD Biosciences cat # 611113), rabbit anti-ERK1/2 (Santa Cruz Biotechnology cat # sc-94), mouse anti-MAP2B (BD Transduction Laboratories cat # 610460), rabbit anti-phospho-Ser637 Drp1 (Cell Signaling Technology cat # 6319), rabbit anti-GFP (Abcam cat # ab290), mouse anti-GFP (clone N86/8, NeuroMab cat# 75-131), mouse anti-FLAG (M2) (Sigma, cat # F3165), mouse anti-V5 (Invitrogen cat # 46-0705), mouse anti-MTCO2 (Neomarkers cat # MS-1372-P), Hoechst 33342 (Invitrogen), Alexa fluorophore-coupled secondary antibodies (Invitrogen), goat anti-mouse IRDye® 680RD (Licor, Cat# 926-68070), goat anti-rabbit IRDye® 800CW (Licor, Cat# 926-32 211), goat anti-rat IRDye® 800CW (Licor, Cat# 926-32219), species-specific horseradish peroxidase (HRP)-conjugated secondary antibodies (PerkinElmer). Other reagents used in this study include: LipoFectamine 2000 (Invitrogen cat #11668-019, forskolin (MP Biomedicals cat # 219066925), rolipram (Enzo Life Sciences cat # BMLPD1750050), doxycycline (Research Products International cat # T17000). HaloTag TMR ligand (Promega cat # G8251), 7-Bromo-1-heptanol (Alfa Aesar cat # H54762), EZview Red anti-FLAG M2 affinity gel (Sigma cat # F2426), GFP nanobody agarose resin (UI biomedical research store cat # 143093). All other reagents were obtained from Sigma.

### Plasmids and mutagenesis

Expression plasmids encoding PPP2R2B/Bβ2 (NM_181676.3) with C-terminal FLAG-GFP tags and RR168EE (monomeric), SSS20AAA (mitochondrial), and SS21DD (mutations), and EE-epitope tagged Aα were described previously [7, 8, 19]. The 15 missense mutations characterized here were introduced into the human Bβ2 coding sequence (NM_181676.3) as per the QuikChange protocol (Invitrogen). The sequence nomenclature of the missense mutations is based on the ClinVar reference isoform Bβ1 (NM_181675.4). For the NanoBiT complementation and HaloTag pulse-chase experiments, the GFP coding sequence was replaced with PCR-amplified LgBiT and HaloTag coding sequences, respectively. The plasmid expressing the human catalytic Cα subunit was modified by replacing the N-terminal 3× hemagglutinin (HA)-tag with SmBiT. Flexible linkers (20-36 residues) were interposed to facilitate conformation-independent interactions between SmBiT and LgBiT.

### Cell culture and transfection

COS-1 cells were cultured in Dulbecco’s Modified Eagle Medium (DMEM, Gibco) supplemented with 10% fetal bovine serum (FBS, Atlanta Biologicals, cat # S11150), and 1% (v/v) GlutaMax (Gibco, cat #35050061) at 37°C with 5% CO_2_. Cells were grown to 60% confluency on collagen-coated plates and transiently transfected using Lipofectamine 2000 (Invitrogen) following the manufacture’s protocol for adhered cells. Immunoprecipitation of Bβ2-FLAG-GFP was carried out using FLAG(M2)-agarose as described [17]. Primary hippocampal neurons were prepared from embryonic day 18 (E18) Sprague Dawley rats (Envigo) as described previously [12, 28]. Cultures were transfected at 9 days in vitro using 0.25 μg of DNA/0.5 μl of NeuroMag (Oz Bioscience Cat# NM51000)/well. For microscopic analysis, cells were grown on #1 cover glass-bottomed 4- or 8-well chambers (Cellvis). Doxycyline inducible PP2A/Bβ2 replacement HEK293T cell lines were generated as previously described for PP2A/B’δ cells [17].

### NanoBit live cell protein interaction assay

COS-1 cells were transfected in 96-well black-walled, clear-bottom plates with equal mass ratios of plasmids expressing SmBiT-Cα, Bβ2-LgBiT, and Aα (see Extended Materials and Methods). After 24–36 h, Nano-Glo® Live Cell Substrate (Promega, Cat. #N2012) was diluted into DMEM with 1% FBS/10 mM HEPES. Growth media was replaced with the live cell-substrate containing media and luminescence was measured using a BioTek Cytation 5 plate luminometer. To normalize for Bβ2-LgBiT expression, a custom-made cell-permeable HiBit peptide (RRRRRRRRRGVSGWRLFKKIS) was added at 0.3 μg/ml to allow for maximum light output.

### HaloTag turn-over assay

Pulse-chase HaloTag-based turnover assay was performed as previously reported [18]. Briefly, COS-1 cells were transfected with Bβ2 (WT/mutant) subunits with C-terminal fusions of HaloTag. After 24–36 h, cells were incubated with 50 nM fluorescent cell-permeant HaloTag® TMR ligand (Promega) for 30 min at 37°C to allow maximal labeling. Further TMR-labeling was blocked by replacing the media with TMR-containing media with 10 μM 7-bromoheptanol (7-Br) blocker (Alfa Aesar) at indicated times. The blocking was terminated by addition of Laemmli buffer and proteins were separated by SDS-PAGE. TMR fluorescence was visualized by Sapphire Biomolecular Imager using a Cy3 filter (Azure Biosystems). Subsequently, total Bβ2 protein was detected by probing the same blot for FLAG tag (also present in Bβ2-HaloTag) using mouse anti-flag antibody and infrared fluorophore-coupled secondary antibody. Relative Bβ2 protein level was determined by dividing TMR fluorescence signal to total protein and normalizing to the zero-time point (set to 1). A one-phase decay model was calculated in GraphPad Prism to determine half-lifes (t_1/2_).

### Immunoprecipitation

COS-1 cells were seeded in collagen-coated 6-well plates at a density of 4 × 10^5^ cells/well. After 2–4 h, cells were transfected with 4 μl/well of Lipofectamine2000 and 2 μg/well of DNA (FLAG tagged Bβ2 (WT/mutants). After 40–48 h, cells were washed with 1X Dulbecco’s phosphate-buffered saline (DPBS) (Gibco) and harvested in 350 μl of immunoprecipitation (IP) lysis buffer (20 mM Tris pH 7.5, 150 mM NaCl, 1% Triton X-100, 1 mM EDTA, 1 mM EGTA, 1 μg/ml leupeptin, 1 mM benzamidine and 1 mM phenylmethylsulfonyl fluoride). Lysates were then centrifuged at 13 000 rpm in 4°C for 15 min. The cell pellet was sonicated with 40 μl of 1X sample buffer. 40 μl of supernatant was collected as “input” and 12.5 μl of 4X sample buffer (200 mM Tris pH 6.8, 40% glycerol, 8% sodium dodecyl sulfate, 0.1% bromophenol blue, 20% β-mercaptoethanol) was added to it. The remaining supernatant was incubated with 20 ul of GFP-nanbody agarose resin (University of Iowa Biomedical Research Store) or EZ view Red ANTI-FLAG M2 affinity gel (Sigma) and rotated for 2 h at 4^o^ C. After 2 h, the immunoprecipitates were centrifuged at 4000 rpm for 2 min at 4^o^ C. 40 μl of the supernatant was collected as the “flowthrough” and 12.5 μl of 4X sample buffer was added to it. The remaining supernatant was aspirated and the immunoprecipitates were washed with IP lysis buffer, rotated for 5–10 min at 4°C and centrifuged at 4000 rpm for 2 min. The immunoprecipitates were washed three more times and extracted in 15 μl of 2X sample buffer. All the samples collected were subjected to western blotting.

### Immunoblotting

Samples were heated at 95°C and equal volume of proteins were loaded onto 10% acrylamide gels and separated at a constant voltage of 180 V for 45 min. The proteins were then transferred onto nitrocellulose membrane at a constant current of 1 Amp for 2 h. The membranes were then washed, stained with Ponceau S and blocked in 2% bovine serum albumin supplemented with 0.05% sodium azide for 30 min at 37°C. The membranes were washed with TTBS (150 mM NaCl, 10 mM Tris pH 7.5, 0.01% Triton X-100) followed by incubation with appropriate primary antibodies overnight at 4°C. On the second day, the membranes were washed with TTBS and incubated with 1:15000-diluted fluorescent dye-conjugated species-specific secondary antibodies (LICOR) for 1 h at room temperature. Subsequently, the membranes were washed with 1X TTBS and visualized using the LICOR Odyssey® Infrared Imaging System. Densitometry analysis was performed using ImageJ. Primary antibodies were prepared at the appropriate dilution in TTBS containing 1% PVP40 and 0.05% sodium azide.

### P‌P2A activity assays

HEK293T cell lines (triclonal = three pooled clones) with inducible PP2A/Bβ2 reduction (RE) was generated for Bβ2 WT, R149P, T246K, N310K and negative control RR168EE as described [17]. Each of the triclonal cell lines were induced with 1 μg/ml of doxycycline for 3 days. Triton X-100 soluble cell lysates (supernatants) were prepared following the immunoprecipitation protocol. Supernatants from each reaction (WT/mutant clonal lines) were incubated with 20 μl of GFP nanobody based agarose resin (University of Iowa Protein and Crystallography Core) rotated for 2 h at 4°C. After 2 h, the immunoprecipitates were centrifuged at 4000 rpm for 2 min at 4^o^ C. Unbound proteins were removed by washing the beads thrice with lysis buffer. After the third wash, phosphatase complexes on agarose beads were resuspended in reaction buffer containing 50 mM Tris (pH 7.5), 0.1% Triton X-100, 2 mM EDTA, 2 mM EGTA, 1 mM benzamidine, 0.05% β-mercaptoethanol, and 2 mg/ml BSA. Bead-bound PP2A complexes were then used to dephosphorylate commercially available phospho-threonine peptide RRA(pT)VA (100 μM) and phosphatase activity was measured using a molybdate/malachite green-based colorimetric assay (Promega). The amount of inorganic phosphate released was measured over time (0–45 min) by absorbance at 630 nm.

### Colocalization of Bβ2 with mitochondria

Hela cells were transfected with GFP-tagged Bβ2 WT or mutants plasmids and fixed with 4% paraformaldehyde 36–42 h later. Cells were then blocked in 4% normal donkey serum in TTBS and incubated overnight at 4°C with 1:1000 dilution of anti-MTCO2 (cytochrome oxidase subunit II, Neomarkers), which stains mitochondria, and anti-GFP290 (Abcam), which stains the GFP-tagged Bβ2. On the next day, the cells were washed with TTBS before incubating with appropriate fluorophore-labeled secondary antibodies and 1 μg/ml Hoechst 33342 to label the nuclei for 1 h at 25°C. Digital images were captured using an epifluorescence microscope (100×, oil immersion lens; DMI4000B; Leica). The colocalization of Bβ2-GFP to mitochondria was determined using custom-written macros and plugins for ImageJ (https://github.com/ststrack/Strack-Lab-software) [29]. Colocalization analysis was preceded by deblurring by iterative deconvolution with a computed point spread function and contrast-limited adaptive histogram equalization (CLAHE).

### Mitochondrial morphology and apoptosis in primary neurons

To assess mitochondrial morphology, primary hippocampal neurons were co-transfected on at 9 days-in-culture with GFP-tagged Bβ2 WT/variants or OMM-targeted-GFP plasmids and a mitochondrial matrix-targeted and V5-tagged mRFP2 plasmid at 4:1 mass ratio. After 72 h, cultures were fixed in 4% paraformaldehyde and blocked in 2% normal donkey serum, followed by overnight incubation at 4°C with primary antibodies directed against GFP and the V5 epitope tag. On the next day, the neurons were washed with TTBS before incubating with the appropriate fluorophore-labeled secondary antibodies and 1 μg/ml Hoechst 33342 for 1 h at RT. 3 × 3 grid of image stacks (~10 focal planes at 0.5 μm intervals) were acquired using the 100× objective of a Keyence BZ-X800E microscope and converted to maximum-intensity Z-projections. The image grid was stitched and haze reduction was performed using Keyence software. A custom-written NIH ImageJ macro (https://github.com/ststrack/Strack-Lab-software) [29] was used to threshold (MaxEntropy) and to analyze images for particle analysis of length and form factor of mitochondria [(4 × π × area)/perimeter^2^]. Mitochondrial morphology was only assessed in healthy neurons, which were identified by intact neurites and ovoid nuclei.

For the neuronal survival assay, primary hippocampal neurons were transfected on days-in-vitro (DIV) 9 with GFP-tagged Bβ2 WT or mutants or OMM-targeted-GFP plasmids. After 72 h (DIV12), cultures were fixed in 4% paraformaldehyde in PBS and blocked in 2% normal donkey serum, followed by overnight incubation at 4°C with primary antibodies directed against GFP and MAP2B. On the next day, the neurons were washed with TTBS before incubating with the appropriate fluorophore-labeled secondary antibodies for 1 h at RT. Cultured neurons were viewed on a Leica DMI4000B epifluorescence microscope using a 20× objective. Neuronal death was assessed as the percentage of transfected neurons (GFP positive) with blebbed or discontinuous dendrites (MAP2B stain) or condensed, irregular or fragmented nuclei. For mitochondrial morphology and neuronal survival assays, experimenters were blinded to transfection conditions.

### Statistics

Data were obtained and analyzed with experimenters blinded to conditions whenever possible. Statistical analyses and generation of graphs was carried out using GraphPad Prism software (version 10.2). Outliers were removed using the ROUT method (Q = 1%). Statistical tests were applied depending on whether data fulfilled requirements for parametric tests and are listed in the legends of each figure. The false positive rate (α) was set at 0.05.

## Supplementary Material

Supplementary_Information_(Sandal_HMG-2024-CE-00490_R1)_ddae166

## Data Availability

Clinical information on *PPP2R2B* variants can be found on ClinVar https://www.ncbi.nlm.nih.gov/clinvar/. *PPP2R2B* isoform expression data (Supplemental Fig. S2C) are available from the Genotype-Tissue Expression (GTEx) Portal at https://gtexportal.org. All other data (Excel spreadsheets, Prism files, images) can be obtained from S.S. upon request.
