# Supplementary material for: De novo missense variants in the PP2A regulatory subunit PPP2R2B in a neurodevelopmental syndrome: potential links to mitochondrial dynamics and spinocerebellar ataxias
Source: Hum Mol Genet. 2024 Nov 20;34(2):193–203. doi: 10.1093/hmg/ddae166 (PMC11780858; doi:10.1093/hmg/ddae166)
Supplement: Supplementary_Information_(Sandal_HMG-2024-CE-00490_R1)_ddae166 [file supplementary_information_(sandal_hmg-2024-ce-00490_r1)_ddae166.docx]

**SUPPLEMENTAL INFORMATION**for Sandal, Jong, et al.

PPP2R2A MA-----------------------------------------------GAGGG------NDIQWCFSQVKGAVDDDVA- 26

PPP2R2B ME-----------------------------------------------EDIDT------RKINNSFLR-----DHSYAT 22

PPP2R2C MG-----------------------------------------------EDTDT------RKINHSFLR-----DHSYVT 22

PPP2R2D MA-----------------------------------------------GAGGGGCPAGGNDFQWCFSQVKGAIDEDVA- 32

fly MGRWGRQSPVLEPPDPQMQTTPPPPTLPPRTFMRQSSITKIGNMLNTAININGAKKPASNGEASWCFSQIKGALDDDVT- 79

worm MVM------EVDEPAVAATTSQNQPQEHANDFDMDTS--------EGPIENDETFEPV--DQINWKFNQVKGNIDADVHT 64

**A40V**

**E37K** **S71L**
PPP2R2A EADIISTVEFNHSGELLATGDKGGRVVIFQQEQENK-IQSHSRGEYNVYSTFQSHEPEFDYLKSLEIEEKINKIRWLPQK 105

PPP2R2B EADIISTVEFNHTGELLATGDKGGRVVIFQREQESK-NQVHRRGEYNVYSTFQSHEPEFDYLKSLEIEEKINKIRWLPQQ 101

PPP2R2C EADIISTVEFNHTGELLATGDKGGRVVIFQREPESK-NAPHSQGEYDVYSTFQSHEPEFDYLKSLEIEEKINKIKWLPQQ 101

PPP2R2D EADIISTVEFNYSGDLLATGDKGGRVVIFQREQENK-SRPHSRGEYNVYSTFQSHEPEFDYLKSLEIEEKINKIRWLPQQ 111

fly DADIISCVEFNHDGELLATGDKGGRVVIFQRDPASK-AANPRRGEYNVYSTFQSHEPEFDYLKSLEIEEKINKIRWLQQK 158

worm EADVISCVEFSHDGEYLATGDKGGRVVIFQRDQSGKYVKGVRSREYNVYSTFQSHEPEFDYLKSLEIDEKINQIRWLKKK 144

**E122K R149P P164S**

PPP2R2A NAAQFLLSTNDKTIKLWKISERDKRP--EGYNLKEEDGRYRDPTTVTTLRVPVFRPMDLMVEASPRRIFANAHTYHINSI 183

PPP2R2B NAAYFLLSTNDKTVKLWKVSERDKRP--EGYNLKDEEGRLRDPATITTLRVPVLRPMDLMVEATPRRVFANAHTYHINSI 179

PPP2R2C NAAHSLLSTNDKTIKLWKITERDKRP--EGYNLKDEEGKLKDLSTVTSLQVPVLKPMDLMVEVSPRRIFANGHTYHINSI 179

PPP2R2D NAAHFLLSTNDKTIKLWKISERDKRA--EGYNLKDEDGRLRDPFRITALRVPILKPMDLMVEASPRRIFANAHTYHINSI 189

fly NPVHFLLSTNDKTVKLWKVSERDKSF--GGYNTKEENGLIRDPQNVTALRVPSVKQIPLLVEASPRRTFANAHTYHINSI 236

worm NAANFILSTNDKTIKLWKISERERKIGDDAWNLPRTN-RINTSSFRGRLQIPSIVPMELIVEASPRRVYGNAHTYHVNSI 223

**T246K**

PPP2R2A SINSDYETYLSADDLRINLWHLEITDRSFNIVDIKPANMEELTEVITAAEFHPNSCNTFVYSSSKGTIRLCDMRASALCD 263

PPP2R2B SVNSDYETYMSADDLRINLWNFEITNQSFNIVDIKPANMEELTEVITAAEFHPHHCNTFVYSSSKGTIRLCDMRASALCD 259

PPP2R2C SVNSDCETYMSADDLRINLWHLAITDRSFNIVDIKPANMEDLTEVITASEFHPHHCNLFVYSSSKGSLRLCDMRAAALCD 259

PPP2R2D SVNSDHETYLSADDLRINLWHLEITDRSFNIVDIKPANMEELTEVITAAEFHPHQCNVFVYSSSKGTIRLCDMRSSALCD 269

fly SVNSDQETFLSADDLRINLWHLEVVNQSYNIVDIKPTNMEELTEVITAAEFHPTECNVFVYSSSKGTIRLCDMRSAALCD 316

worm SVNSDQETFLSADDLRVNLWNLEITNESFNIVDIKPANMEELTEVITAAEFHPTQCNWFVYSSSKGSIRLCDMRDRALCD 303

**P271R
 R274G N310K**

PPP2R2A RHSKLFEEPEDPSNRSFFSEIISSISDVKFSHSGRYMMTRDYLSVKIWDLNMENRPVETYQVHEYLRSKLCSLYENDCIF 343

PPP2R2B RHTKFFEEPEDPSNRSFFSEIISSISDVKFSHSGRYIMTRDYLTVKVWDLNMENRPIETYQVHDYLRSKLCSLYENDCIF 339

PPP2R2C KHSKLFEEPEDPSNRSFFSEIISSVSDVKFSHSGRYMLTRDYLTVKVWDLNMEARPIETYQVHDYLRSKLCSLYENDCIF 339

PPP2R2D RHSKFFEEPEDPSSRSFFSEIISSISDVKFSHSGRYMMTRDYLSVKVWDLNMESRPVETHQVHEYLRSKLCSLYENDCIF 349

fly RHSKQFEEPENPTNRSFFSEIISSISDVKLSNSGRYMISRDYLSIKVWDLHMETKPIETYPVHEYLRAKLCSLYENDCIF 396

worm AYAKIFEEPEDPQSRSFFSEIIASVSDVKFSHNGRYLLTRDYLTVKVWDLNMESQPVETYPVHNYLRTKLCALYENDSIF 383

**D372H**

PPP2R2A DKFECCWNGSDSVVMTGSYNNFFRMFDRNTKRDITLEASRENNKPRTVLKPRKVCASGKRKK-----------DEISVDS 412

PPP2R2B DKFECVWNGSDSVIMTGSYNNFFRMFDRNTKRDVTLEASRENSKPRAILKPRKVCVGGKRRK-----------DEISVDS 408

PPP2R2C DKFECAWNGSDSVIMTGAYNNFFRMFDRNTKRDVTLEASRESSKPRAVLKPRRVCVGGKRRR-----------DDISVDS 408

PPP2R2D DKFECCWNGSDSAIMTGSYNNFFRMFDRDTRRDVTLEASRESSKPRASLKPRKVCTGGKRRK-----------DEISVDS 418

fly DKFECCWNGKDSSIMTGSYNNFFRVFDRNSKKDVTLEASRDIIKPKTVLKPRKVCTGGKRKK-----------DEISVDC 465

worm DKFECDWSGDDKHILTGSYHNLFRSYARGNNQDAKTWEARPQ-EPHSQLRSRFVVPSAKRKRNNLSSSGETTEEDLSSDQ 462

**I427T clinically characterized**

PPP2R2A LDFNKKILHTAWHPKENIIAVATTNNLYIFQDKVN---- 447 predicted pathogenic

PPP2R2B LDFSKKILHTAWHPSENIIAVAATNNLYIFQDKVN---- 443

PPP2R2C LDFTKKILHTAWHPAENIIAIAATNNLYIFQDKVNSDMH 447 **surface exposed**

PPP2R2D LDFNKKILHTAWHPVDNVIAVAATNNLYIFQDKIN---- 453 **buried**

fly LDFNKKILHTAWHPEENIIAVAATNNLFIFQDKF----- 499

worm LQFDRKILHTAWHPKDNIIALAATNNLYIFSDV------ 495

Supplemental Figure S1. **Sequence alignment of human, fly, and worm PPP2R2 regulatory subunits**. Variants affect residues highly conserved in human PPP2R2A/B/C/D, as well as their Drosophila melanogaster (twin) and Caenorhabditis elegans (sur-6) orthologs. More information on the seven predicted as pathogenic variants can be found in Supplemental Table S1


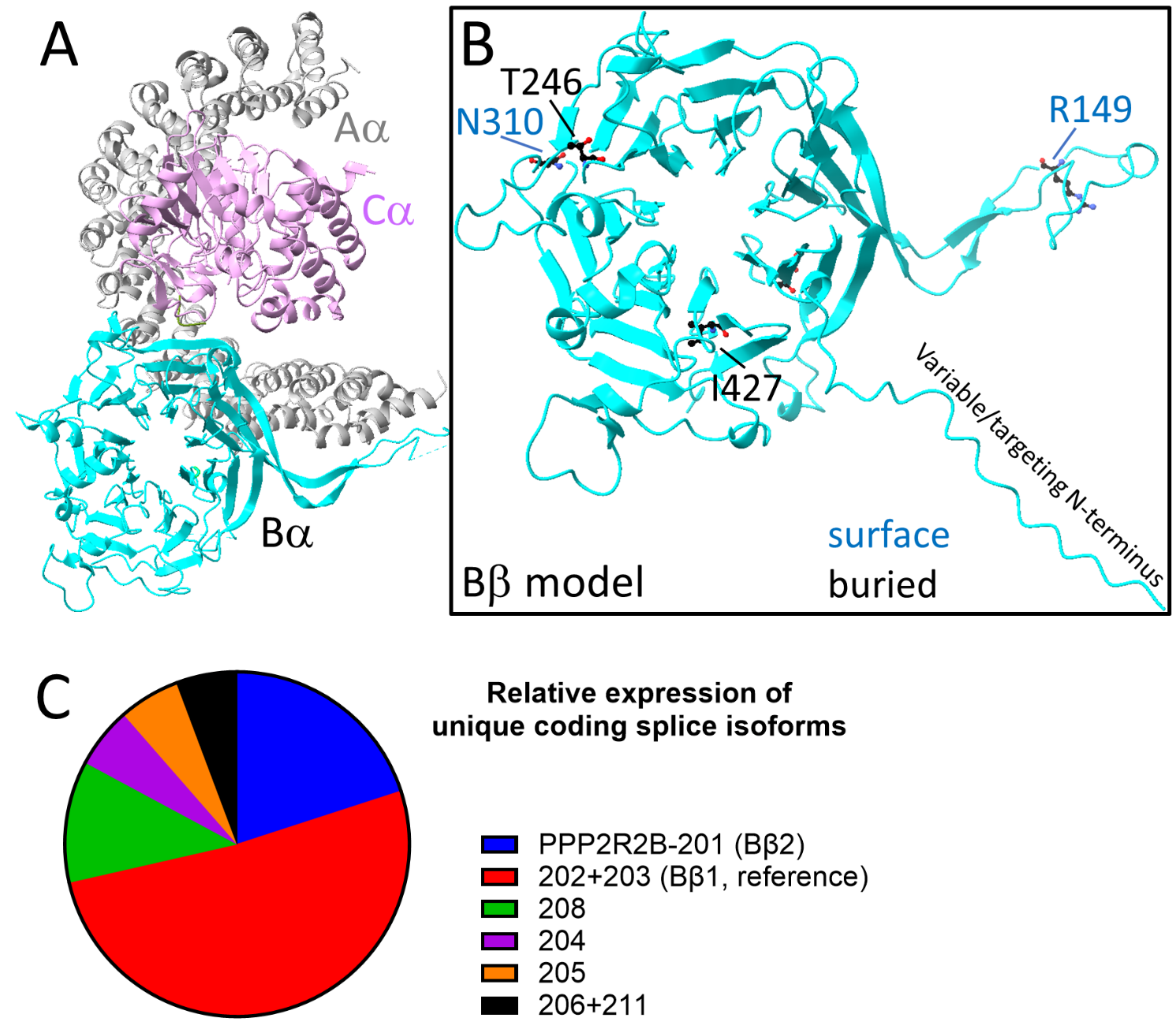


Supplemental Figure S2. **Structural context of PPP2R2B variants and relative isoform expression in the human brain.** (A) Ribbon diagram of the PP2A heterotrimer containing the PPP2R2A/Bα subunit (3DW8) [1]. (B) AlphaFold2-based PPP2R2B/Bβ structure model highlighting residues affected by missense mutations characterized in this report. (C) Relative mRNA expression of coding *PPP2R2B* coding isoforms in the human cortex (BA9) according GTex. Isoform 202 and 203 (ensemble nomenclature) both encode Bβ1 but differ in their 5’UTR. Similarly, mRNA isoforms 206 and 211 encode the same protein.


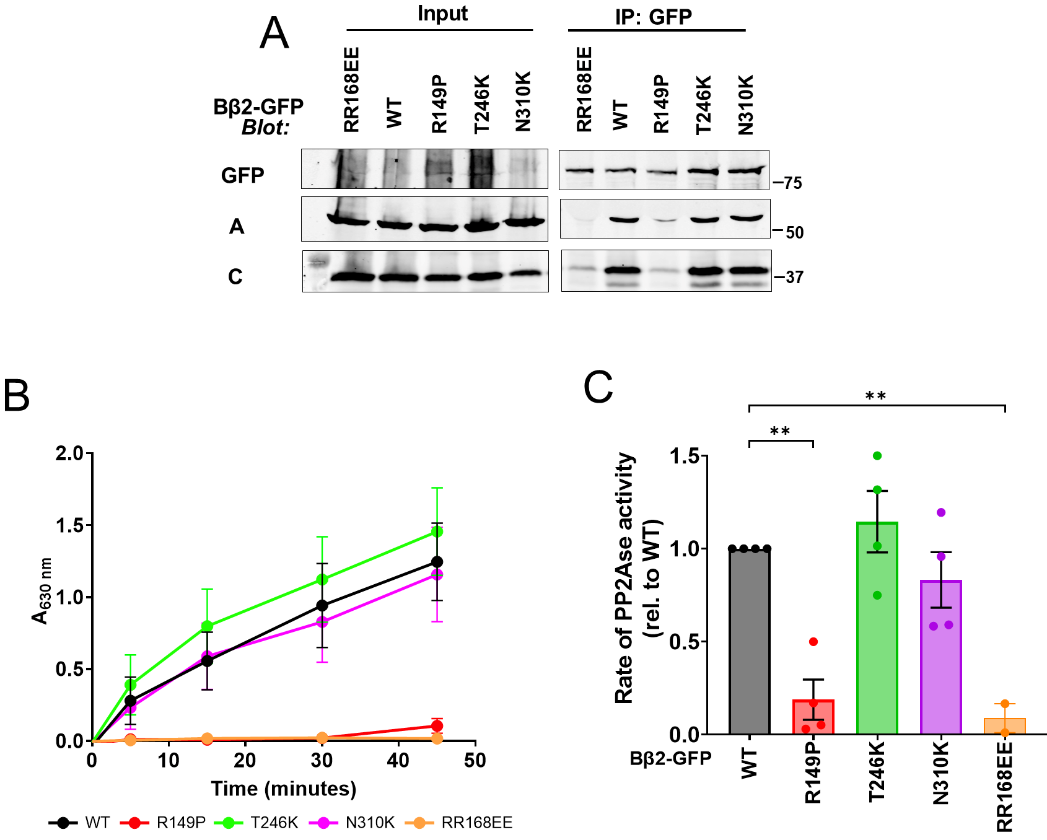


Supplemental Figure S3**. Assays of PPP2R2B-associated phosphatase activity.** (A-C) Bβ2 subunits were analyzed for PP2A catalytic subunit-mediated enzymatic activity via an on-bead based phosphatase assay. (A) Representative immunoblots showing the scaffold A and catalytic C subunits co-isolated via GFP nanobeads from HEK293T lysates inducibly expressing Bβ2-GFP. (B-C) Immuno-isolated PP2A/Bβ2 complexes were tested against a synthetic phospho-threonine peptide substrate (Arg-Arg-Ala-(p)Thr-Val-Ala, Promega PP2A assay kit) and the released phosphate was monitored over time by measuring the absorbance of Malachite green at 630 nm. Empty resin bead background reading was subtracted from each reaction. Shown are dephosphorylation time courses (B) and initial rates (C) for the indicated PP2A/Bβ2 complexes. Activity reflects the abundance of the PP2A C subunit in the Bβ2 complexes. **, p < 0.01 based on Kruskal-Wallis analysis followed by Dunn’s *post hoc* test.

**
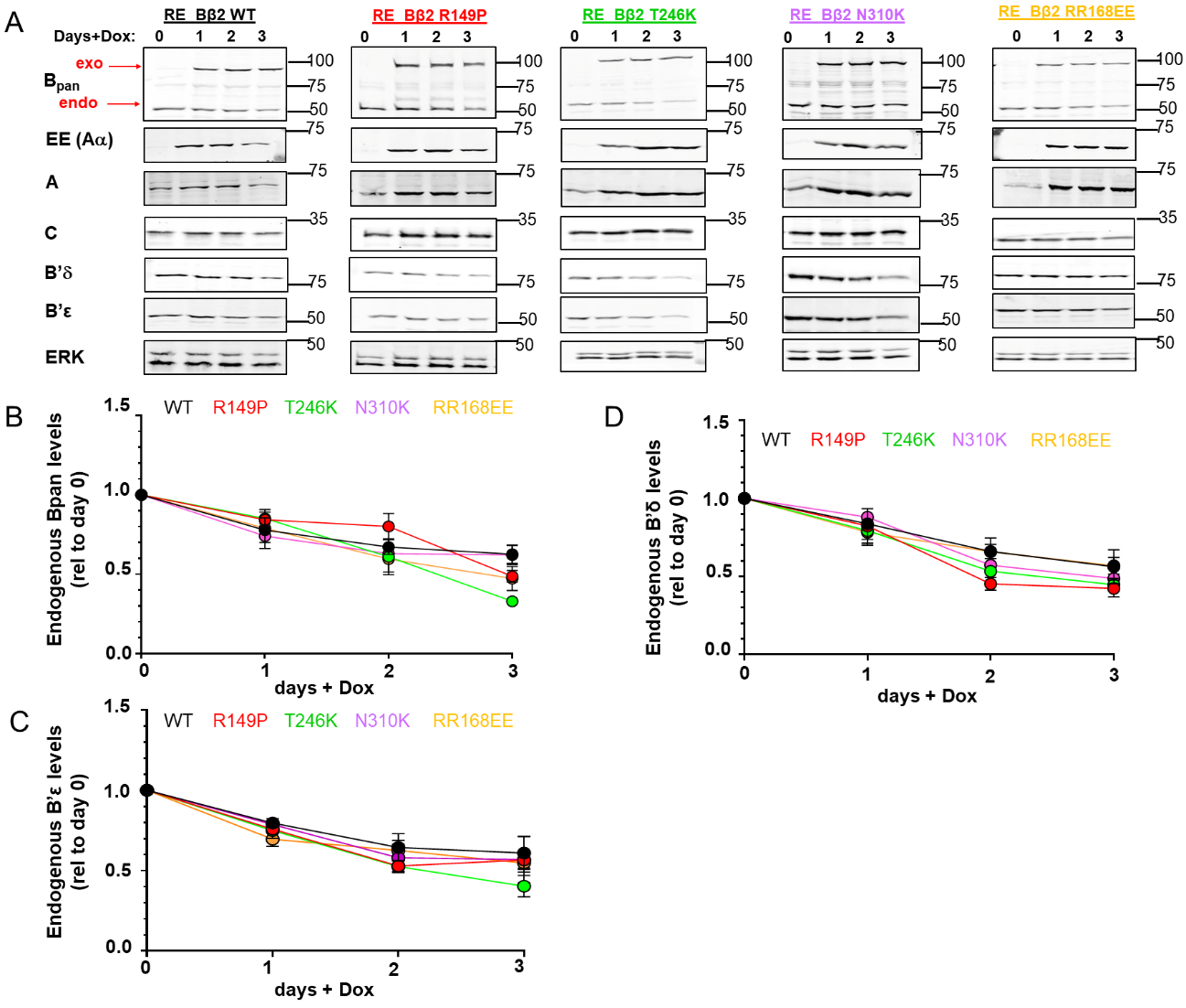
**

Supplemental Figure S4**. Characterization of inducible PP2A/Bβ2 reduction cell lines.** (A-C) Clonal HEK293T cell lines with inducible PP2A/Bβ2 WT, R149P, T246K, N310K and RR168EE were treated with 1 ug/mL of doxycycline for the indicated number of days before immunoblotting for the indicated proteins. (A) Representative western blots with the positions of molecular mass markers (in kDa) indicated on the right. (B-D) Quantification of endogenous B (pan-specific antibody, B), B’δ (C) and B’ε (D) levels normalized to ERK and day 0. Data shown are means ± SEM of three clonal cell lines.

| **Variant (NM_181675.4)** | **Accession** | **Reference** | **Frequency** | **Condition(s)** | **Pathogenicity** |
| --- | --- | --- | --- | --- | --- |
| p.Ala40Val | 548555 |  | 4.78871E-06 | Spinocerebellar ataxia type 12 | 0.993 |
| p.Asp372His | 2662862 |  | Absent | not provided | 0.991 |
| p.Arg149Pro | 2503290 | Hamdan, 2014 | Absent | not provided | 0.989 |
| p.Glu122Lys | 2589453 |  | 1.79681E-05 | Inborn genetic diseases | 0.987 |
| p.Arg274Gly | 2661939 |  | Absent | not provided | 0.985 |
| p.Thr246Lys | 1712310 | this report | Absent | Spinocerebellar ataxia type 12 | 0.946 |
| p.Ser71Ile | 2528415 |  | Absent | Inborn genetic diseases | 0.937 |
| p.Pro271Arg | 2477461 |  | 4.10551E-06 | Inborn genetic diseases | 0.91 |
| p.Glu37Lys | SUB14519904 | this report | Absent | Spinocerebellar ataxia type 12 | 0.793 |
| p.IleI427Thr | 1711732 | this report | Absent | Global developmental delay | 0.771 |
| p.Pro164Ser | 2239637 |  | 6.84166E-07 | Inborn genetic diseases | 0.578 |
| p.Asn310Lys | 1319957 | this report | Absent | Neurodevelopmental disorder | 0.527 |

Supplemental Table S1. **Additional predicted pathogenic *PPP2R2B* missense variants.** The table lists variants characterized here, as well additional unreported, but predicted pathogenic variants identified by cross-referencing ClinVar and gnomAD. Variants were ranked according to pathogenicity scores predicted by AlphaMissense [2]. Only missense variants with scores greater than 0.5 are listed. “Absent” in the Frequency column indicates variants listed in ClinVar, but not gnomAD (4.1.0).

**References**

1. Xu Y, Chen Y, Zhang P, Jeffrey PD, Shi Y. Structure of a protein phosphatase 2A holoenzyme: insights into B55-mediated Tau dephosphorylation. *Mol. Cell* 2008; **31**: 873-885.

2. Cheng J, Novati G, Pan J, Bycroft C, Zemgulyte A, Applebaum T, Pritzel A, Wong LH, Zielinski M, Sargeant T *et al.* Accurate proteome-wide missense variant effect prediction with AlphaMissense. *Science* 2023; **381**: eadg7492.
